# Surgical Treatment of Peri-Implant Defects with L-PRF-Xenograft Bone Blocks: A Prospective Case Series

**DOI:** 10.3390/bioengineering13030328

**Published:** 2026-03-12

**Authors:** Orlando Martins, Ana Messias, Isabel Baptista, Sérgio Matos

**Affiliations:** 1 Institute of Periodontology, Faculty of Medicine, University of Coimbra, Av. Bissaya Barreto, Blocos de Celas, 300-075 Coimbra, Portugal; isabelpoiaresbaptista@gmail.com (I.B.); sergiomatos1@sapo.pt (S.M.); 2 Institute of Medicine and Oral Surgery, Faculty of Medicine, University of Coimbra, Av. Bissaya Barreto, Blocos de Celas, 300-075 Coimbra, Portugal; 3 Institute of Implantology and Prosthodontics, Faculty of Medicine, University of Coimbra, Av. Bissaya Barreto, Blocos de Celas, 3000-075 Coimbra, Portugal; ana.messias@uc.pt; 4 Center of Mechanical Engineering Materials and Processes (CEMMPRE), Departamento de Engenharia Mecânica, University of Coimbra, 3030-788 Coimbra, Portugal

**Keywords:** peri-implantitis, reconstructive, L-PRF membrane, xenograft, L-PRF block, peri-implant defect

## Abstract

The goal of this paper was to determine the efficacy of the surgical treatment of two-wall peri-implant defects filled with L-PRF/xenograft block in the reduction of peri-implant marginal bone levels after 12 months. Ten patients with two-wall peri-implant defects were included. Patients received presurgical treatment followed by a surgical reconstructive intervention with bone blocks obtained by mixing bovine origin xenogeneic bone graft grains with L-PRF membranes. Patients were followed up at 3, 6, 9, and 12 months for oral hygiene and disease relapse evaluation and assessment of the primary outcome marginal bone levels (MBL) and clinical outcome variables such as probing depth (PD), bleeding on probing (BOP), and mucosal recession. Data was analyzed for changes between baseline and the 6- and 12-month follow-ups. Mean MBL was 5.1 ± 1.7 mm and 1.58 ± 0.92 mm at baseline and 6 months after the procedure, indicating a statistically significant decrease (*p* = 0.0005). At 12 months post-surgery, marginal bone levels remained stable at 1.8 ± 0.9 mm, with no statistically significant difference from the previous evaluation (*p* > 0.05). From baseline to 6 months there was also a statistically significant decrease in PD (from 8.07 ± 1.51 mm to 3.33 ± 0.59, *p* < 0.0001) and BOP (from 60.0% to 13.0% of affected surfaces, *p* < 0.001). No changes were observed from the intermediate to the 12-month follow-up (*p* > 0.05 for all variables).

## 1. Introduction

Oral rehabilitation using dental implants is considered a safe and predictable treatment for both partially and edentulous patients and is associated with high survival rates [1,2]. More than 12 million implants are placed annually worldwide [3]. Although survival may be used as an outcome to assess implant function, it does not give attention to biological and/or esthetic status of the surrounding tissues. In fact, implants may be functional but still be associated with inflammation and pathological bone loss, i.e., having a positive survival outcome but not fulfilling the definition of healthy peri-implant tissues [4].

Biological complications affecting peri-implant tissues are a subject of significant contemporary debate. Peri-implantitis (PI) is defined as a “pathological condition occurring in tissues around dental implants, characterized by inflammation in the peri-implant connective tissue and progressive loss of supporting bone” [5]. Recently, PI, in the absence of initial radiographs, has been characterized by radiographic evidence of bone level ≥ 3 mm and/or probing depth (PD) ≥ 6 mm in conjunction with profuse bleeding [6]. If left untreated, peri-implantitis may progress in a nonlinear, accelerating pattern, destroying the implant-supporting bone and ultimately lead to implant loss [7]. Patients with a history of chronic periodontitis, poor plaque control, and no regular maintenance care after implant therapy have an increased risk of developing peri-implantitis [5].

Peri-implantitis treatment includes several therapeutic approaches, such as pocket elimination and access flap and reconstructive procedures. Access flap and pocket elimination surgery results suggest a high degree of heterogeneity. Disease recurrence occurred frequently, and implant loss is not uncommon [8]. The evidence on regenerative treatment of peri-implantitis related defects is limited [9,10]. Regardless of the surgical technique employed, the resolution of peri-implantitis remains challenging and unpredictable [11]. Regenerative treatment of peri-implantitis using autologous bone grafts, bone substitute materials alone or in combination with membranes, titanium porous particles, or even biologic agents has already been investigated. Among biological agents, leucocyte-platelet-rich fibrin (L-PRF) has been widely used in periodontology, implantology, and oral surgery [12]. Its fibrin architecture and leucocyte content play a central biological role [13,14,15]. The strong fibrin polymerization and dense fibrin network influence the biology of the material and the cells it entraps, enabling the sustained release of significant amounts of growth factors and matrix proteins during more than 7 days. Leucocytes appear to be a major source of these growth factors [16]. Recently, Cortellini et al. investigated the radiographic and clinical outcome of a novel guided bone regeneration technique using a L-PRF-xenograft block, thereby establishing proof-of-concept for this new tissue engineering technique [17].

Notwithstanding this, clinical studies validating and determining the efficiency of this technique are still missing in the literature, particularly for peri-implant defects. Therefore, the main objective of the present study is to describe the surgical treatment of biologically demanding two-wall peri-implant defects filled with L-PRF/xenograft block after decontamination with a glycine air-flow system, and to determine the efficacy of the technique in reducing peri-implant marginal bone levels after 12 months. This article is reported in accordance with the STROBE (Strengthening the Reporting of Observational Studies in Epidemiology) guidelines.

## 2. Materials and Methods

The present study was designed as a prospective case series study carried out at the Department of Dentistry of the Faculty of Medicine of the University of Coimbra and was approved by the Ethical Committee of the institution (CE-028/2019). During the recruitment appointment, all participants received a thorough explanation of the study protocol. Each participant signed an informed consent form in accordance with the Declaration of Helsinki (1975, revised 2008) and was informed of treatment alternatives.

### 2.1. Patient Selection

Patient selection was conducted between March 2019 and December 2019. Patients were included in this prospective study if they met the following inclusion criteria: (1) presence of peri-implantitis on at least one implant, defined as the presence of radiographic evidence of bone levels ≥ 3 mm and/or probing depths ≥ 6 mm in conjunction with profuse bleeding [6]; (2) no active periodontitis; (3) patients referred at the Dentistry Department of the Medical Faculty of Coimbra’s University; (4) non-smoker or smoker < 10 cig/day; (5) implants had to be in function for more than 12 months; (6) willingness to commit to a 1-year follow-up.

During surgery, patients were assessed for the following secondary inclusion criteria: presence of a two-wall peri-implant defect. Exclusion criteria were as follows: (1) systemic disease or condition as a potential absolute contra-indication to surgical treatment; (2) immunosuppressed or immunocompromised patients; (3) presence of uncontrolled or poorly controlled diabetes; (4) pregnancy and lactation; (5) use of medication that induces gingival hyperplasia; (6) smoker ≥ 10 cig/day; (7) acute infection (abscess) in the site intended for treatment; (8) implant mobility; (9) implants previously surgically treated for peri-implantitis; (10) level of peri-implant bone loss > 75%; (11) poor oral hygiene and motivation defined as a full mouth plaque score (FMPS) > 20% and a full mouth marginal bleeding score (FMMBS) > 20%.

### 2.2. Peri-Implant Clinical Examination

The clinical examination comprised the systematic collection of probing depth (PD), clinical attachment level (CAL), and mucosa recession (MR). These parameters were assessed at baseline and at 6 and 12 months post-surgery. Bleeding on probing (BOP) and suppuration (SUP) were assessed 30 s after probing. With the exception of KT, all parameters were assessed at six sites/implant. The amount of keratinized tissue (KT) was measured at the centro-buccal site of each implant. All measurements were performed by a single periodontologist calibrated examiner (OM). Intraexaminer calibration was determined by taking repeated measurements of the same peri-implant pockets until reaching a high degree of repeatability (90% agreement within 1 mm). Clinical measurements were taken with a PCP-15 periodontal probe (Hu-Friedy, Chicago, IL, USA) with light pressure at six sites per implant, which were rounded to the nearest 0.5 mm.

### 2.3. Peri-Implant Radiographic Examination

Intraoral periapical radiographs were obtained immediately before surgery and at 6 and 12 months post-surgery using a customized film holder and a long-cone-equipped dental X-ray unit. Marginal bone level (MBL) was measured from the implant shoulder to the first bone-to-implant contact using a specific software (ImageJ^®^ 1.52p, NIMH, Bethesda, MD, USA) at both the mesial and distal areas of the implant. The mean value of these two measurements was used for statistical analysis. Implant length or known dimension of implant threads were used as reference for calibration. The images were analyzed by two periodontologists with a high degree of experience in radiographic analysis (IPB and DSS), and they were not involved in any other step of this study. If inter-examiner results were 0.1 mm or less, the mean of both measurements was used. If it was >0.1 mm, the investigators reanalyzed the image together to reach a consensus [18].

### 2.4. Presurgical Phase

Prior to surgery, all patients underwent a thorough periodontal examination, including motivation and oral hygiene instructions, as well as multiple sessions of supra and subgingival instrumentation, if needed, until reaching acceptable levels of plaque control (FMPS < 20%) and inflammation control (FMMBS < 20%). Before the initiation of peri-implantitis treatment, all included patients with periodontitis had their pathology stabilized. All patients underwent non-surgical peri-implantitis treatment. After suprastructure removal, peri-implant debridement was performed with a titanium curette (Deppeler^®^, Rolle, Switzerland) and subsequent subgingival debridement with an air-flow system with glycine (Perio Mate, NSK^®^, Kanuma, Japan). Finally, local subgingival irrigation using 5 mL of 10% povidone–iodine (Betadine^®^, Meda Pharma, Lisbon, Portugal) was performed. This non-surgical treatment was performed every three days for a period of two weeks, starting four weeks prior to the surgical phase and concluding two weeks before surgery.

### 2.5. L-PRF Preparation

L-PRF preparation was performed according to a previously described original protocol [19,20]. Venipuncture was executed for blood collection into eight sterile tubes (9 mL/tube), which were immediately centrifuged at 2700 rpm/400 g RCF for at least 12 min using a table centrifuge (Intra-Spin system, Intra-Lock, Boca Raton, FL, USA). After 3 min, the white tubes were removed, and the remaining six were left for 9 more min. The liquid fibrinogen was collected from 2 tubes using a plastic syringe and kept. After centrifugation, L-PRF clots were removed from the six tubes, separated from the red element phase with pliers, placed in the specific PRF box, and compressed until L-PRF membranes were obtained. Two L-PRF membranes were chopped and mixed with 0.5 g of xenograft (Creos^®^ Xenogain, S, bowl, Nobel Biocare, Zurich, Switzerland). Liquid fibrinogen was added, and the mixture was gently shaped to the desired form to obtain an L-PRF bone block ready to use.

### 2.6. Therapeutic Procedure

**Figure 1:**
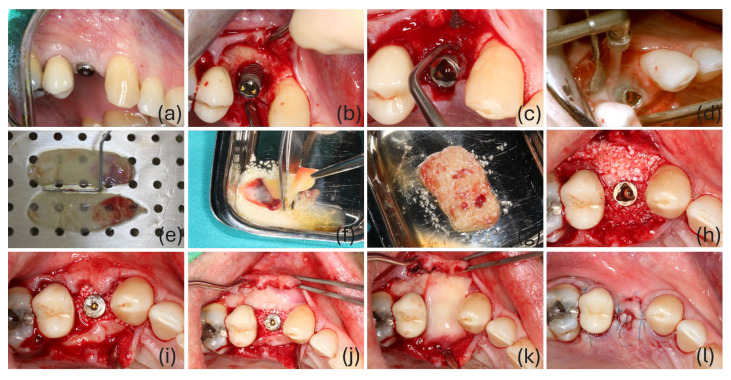
Representative case of the surgical treatment of two-wall peri-implant defects with L-PRF bone blocks. (**a**) Initial situation; (**b**) peri-implant defect; (**c**) implant surface debridement with titanium curette; (**d**) implant surface debridement with air-flow; (**e**) L-PRF membranes; (**f**) chopping of the L-PRF membrane to mix with xenograft; (**g**) L-PRF block (L-PRF membranes with xenograft); (**h**) occlusal view of the L-PRF block placed in the peri-implant defect; (**i**) L-PRF membrane covering the palatal L-PRF block; (**j**) occlusal view of both buccal and palatal L-PRF membranes; (**k**) occlusal placement of the L-PRF membrane over the the peri-implant defect. (**l**) occlusal view of the final suture.

All surgeries were performed by the same experienced periodontologist (OM). Suprastructures were removed prior to surgery and replaced with transmucosal healing abutments. Intrasulcular incisions were made, avoiding vertical releasing incisions in the flap design whenever possible, and a full-thickness mucoperiosteal flap was elevated. Pocket epithelium and granulation tissue were removed using a microsurgery BW002 blade (MJK^®^, Asnières-sur-Seine, France) and titanium curettes (Deppeler^®^, Switzerland), respectively. The implant surface was rinsed with saline solution and then debrided with a titanium curette to remove calculus. The implant surface was cleaned using an air-flow system with glycine (Perio Mate, NSK^®^, Japan). Finally, the implant surface and the peri-implant defect were rinsed with L-PRF exudate. A L-PRF membrane was placed covering the buccal site of the defect, and another was placed to cover the lingual/palatal site. Both membranes were positioned to correspond to the areas of the peri-implant defect lacking a bony wall. The peri-implant defect, between membranes, was filled with the L-PRF bone block. Finally, two additional membranes were placed over the occlusal area. The flaps were sutured with 5/0 propylene suture (Prolene^®^, Ethicon, Somerville, NJ, USA) (Figure 1). All implants were left submerged, except those in the aesthetic area or supporting a dolder bar (Table 1). Patients were given instructions to take amoxicillin 1 g (12-12 h; 7 days), ibuprofen 600 mg (12-12 h; 5 days), and paracetamol 1 g (8-8 h; SOS).

### 2.7. Follow-Up

Sutures were removed two weeks post-surgery. Patients were evaluated at 1, 2, and 4 weeks after surgery and subsequently recalled at 3, 6, 9, and 12 months. During the follow-up visits, all patients received oral hygiene instructions, and, when necessary, tooth cleaning and bimaxillary polishing were performed. Outcome variables assessed at month 6 and 12 included PD, BOP, CAL, MR, and MBL. All implants left submerged were exposed about five months after surgery and rehabilitated with the corresponding prosthetic components.

### 2.8. Outcomes

The primary outcome for the present study was the change of marginal bone level from the time of surgery to the 12-month follow-up. Marginal bone level was measured from the implant shoulder to the first visible bone-to-implant contact using a dedicated software (ImageJ^®^, NIMH, USA) at both the mesial and distal areas of the implant. Implant length or known dimension of implant threads were used as reference for calibration. Secondary outcome measures were successful treatment of peri-implantitis or disease relapse, defined as probing pocket depth reduction, resolution, or reduction of BOP/suppuration and stabilization of marginal bone levels or even bone regeneration [21]. The clinical parameters assessed were probing depth, bleeding on probing, clinical attachment level, mucosal recession, and keratinized tissue.

### 2.9. Sample Size

The determination of the number of patients to include was performed considering that this study was designed as a pre–post evaluation of the marginal bone level resulting from the reconstructive intervention. Calculations were performed in G-Power Version 3.1.9.6 and assumed a normally distributed mean gain of 2 mm from surgery to 12 months post-surgery, with a 2 mm standard deviation, corresponding to an effect size d of 1, similar to the results by Khoshkam et al. and Chan et al. [9,22]. At 80% power (beta = 0.2) and a significance level of alpha = 0.05, a priori sample size calculations determined that 10 patients were required to detect the aforementioned bone level change.

### 2.10. Statistical Analysis

Outcome variables were descriptively analyzed as mean and standard deviation (continuous variables) or count and relative frequency (categorical variables). Changes between baseline and 6- and 12-month follow-ups were analyzed using the Friedman test and pairwise comparisons with Bonferroni correction. Statistical analyses were executed using RStudio “Chocolate Cosmos” Release (packages rstatix, ggplot2 and ggpubr) with a preset significance level of *p* < 0.05.

## 3. Results

### Participants

After screening 15 potentially eligible patients,10 patients (5 male and 5 female) with a mean age of 49.0 ± 8.0 years were included in the present study. For patients who had more than one peri-implant defect meeting the inclusion criteria, only the most severe was included in this study (Table 1). One patient smoked 8 cig./day and another stopped smoking on the day of surgery (#5). All patients presented normal healing without pronounced pain, major inflammatory reactions, or swelling. Only one patient (#8) reported minor discomfort due to swelling. Two patients presented with submerged healing (#1 and #2). These patients had their prosthetic component placed over the implant 5 months post-surgery.

Table 2 contains the descriptive presentation of radiographic and clinical outcomes throughout this study. The corresponding treatment effects (variation over time) and statistical comparisons are detailed in Table 3.

**Figure 2:**
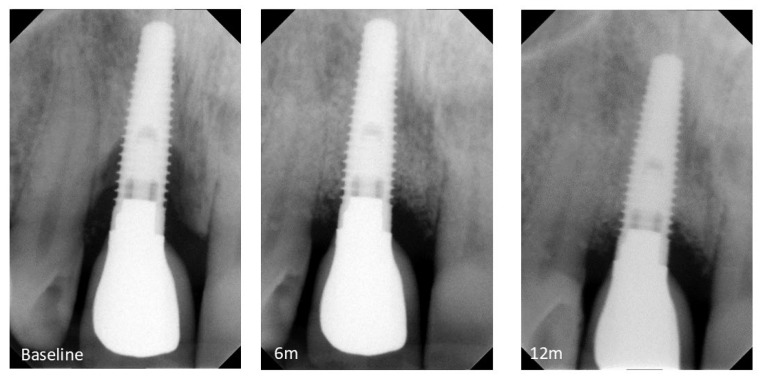
Radiographic follow-up of the reconstructive surgical protocol over a 12-month period (6m: 6 months; 12m: 12 months).

Figure 2 presents a representative sequence of the radiographic evolution over the course of 12 months. At baseline, the mean marginal bone level was 5.1 ± 1.7 mm. The highest absolute value for MBL was 7.225 mm (45.19% of the implant body height), present at the distal site of the implant (patient #5). Several implants had more than 50% of bone loss at the proximal sites. The procedure induced a statistically significant variation of the mean marginal bone levels (*p* = 0.0005). Six months after surgery, all patients had a decrease in the MBL values, and the mean MBL was 1.58 ± 0.92 mm, corresponding to a statistically significant decrease of 3.49 ± 1.17 mm compared to baseline. At 12 months post-surgery, marginal bone levels remained stable at 1.83 ± 0.9 mm, as represented in Table 2, with no statistically significant difference from the previous evaluation.

At baseline, the mean PD was 8.07 ± 1.51 mm, and the deepest site had a PD of 13 mm (patient #2). All patients had profuse bleeding on probing on at least two sites/implant, and the mean BOP value was 60.0 ± 34.0%. However, four patients had a BOP of 100% (#2, #5, #8 and #9). No MR was observed, and only two patients presented suppuration (2.0 ± 5.0%). The mean KT was 4.2 ± 1.75 mm. At the 6-month evaluation, the mean PD was 3.33 ± 0.59 mm, which corresponds to a significant decrease of 4.73 ± 1.54 mm compared to baseline (*p* < 0.0001). Even though some of the implants presented BOP, the final mean value was 13.0 ± 15.0% with a significant decrease of 55.0 ± 39% compared to baseline (*p* = 0.002). At this time point, the mean MR was 0.05 ± 0.08 mm, representing a non-significant variation (*p* > 0.05). No implant presented suppuration. Regarding KT, the mean value was 3.40 ± 1.84 mm (*p* > 0.05).

No significant differences were found for the clinical parameters from the 6-month follow-up to 12 months. The mean PD was 3.53 ± 0.38 mm, which corresponds to a variation of 0.2 ± 0.6 mm from the previous appointment and an overall significant decrease of 4.5 ± 1.3 mm compared to baseline. The highest absolute PD value was 5 mm (patient #9). The mean BOP value remained 13.0 ± 13.0%, indicating the stability of the treatment from 6 months onwards and a significant decrease from baseline (*p* < 0.0001). Three of the treated implants had an MR of 1 mm at the buccal or lingual aspects, but no significant variation was detected from the baseline situation (*p* > 0.05). No implant had suppuration. KT had a mean value of 3.50 ± 1.78 mm (*p* > 0.05).

## 4. Discussion

This observational study aimed to evaluate a surgical approach for peri-implantitis in biologically demanding class I, two-wall defects based on a reconstructive procedure with L-PRF membranes and a bone block of xenograft/L-PRF associated with an implant surface decontamination with glycine air flow, during a 12-month follow-up.

After 12 months of healing, the MBL gain was 3.25 ± 1.32 mm, mean PD decreased 4.53 ± 1.30 mm, and the percentage of BOP was reduced by 55.0 ± 31.0%. Between the 6- and 12-month evaluation, there were no significant changes in all parameters, indicating the medium-term stability of the treatment. Although similar results have been reported in the literature for reconstructive regenerative approaches of peri-implant defects, no other studies have described such results in biologically demanding two-wall defects, as included in the present study. Indeed, the influence of peri-implant defect configuration on the outcomes of reconstructive treatment is well knwon, with Cl Ic, circumferential defects, being the most promising due to its containment anatomy and defects with a buccal dehiscence (Cl Ia) or 2–3 wall configuration (Cl Ib) being considered unfavorable [23,24]. Data from periodontal biology highlights the impact of the absence of bone wall containment on clot and regenerative material stabilization. In those cases, wound revascularization relies primarily on vascular and cellular elements in the periodontal ligament, and synergistic effects with the alveolar bone, while depending on space provision established by the horizontal dimension of the alveolar base [25,26,27]. Consequently, due to the limited regenerative potential of the alveolar bone, peri-implant defect reconstruction may be even more difficult and challenging than in periodontal sites. The current study included only two-wall peri-implant defects and employed a surgical technique with L-PRF block that simultaneously meets two fundamental principles: space-maintenance with a scaffold and physical stabilization of the material. This avoids particle dispersion and allows easy adaptation of the material in these non-containing defects, while providing a matrix that allows cell recruitment, neovascularization, and the delivery of growth factors [28]. These characteristics are particularly important in the absence of a traditional GBR procedure [29], which has provided contradictory results in the literature. Comparative studies using augmentative procedures to treat peri-implantitis with or without resorbable membranes are not unanimous [24,30,31], and it seems that the additional use of a resorbable membrane in addition to a xenograft to treat peri-implantitis defects might not improve the outcome [31]. In addition, the application of a membrane is also costly, time consuming, and technique sensitive [22,32].

Despite the two-wall, non-contained nature of the defects treated in the present study, the significant increase in MBL and a decrease in PD between baseline and 6 months, which is maintained forward to the 12-month evaluation, may be partially explained by the biological behavior of the L-PRF/xenograft block. L-PRF retains substantial amounts of cytokines and growth factors within a three-dimensional fibrin scaffold, which are released over a period of 7–11 days [33,34,35], improving the recruitment and proliferation of a variety of cells like endothelial cells and osteoblasts [36,37]. The surgical approach here described also included covering the filled defect with several layers of buccal, palatal, and occlusal L-PRF membranes in order to increase local release of biological agents and improve wound stabilization to enhance clot stabilization, since this is an essential key for healing [26]. This surgical methodology enabled not only outside angiogenic activity, as provided by the L-PRF membranes, but also angiogenic activity in the inner part of the block due to the presence of L-PRF matrix fragments with intrinsic cellular activity. The clinical benefits of L-PRF are already well established and were confirmed by several systematic reviews, not only on periodontal wound healing [12] but also ridge preservation [38], bone regeneration, and osseointegration [12,39]. Even though the proof-of-concept for L-PRF/xenograft block for bone augmentation was obtained by Cortellini et al. with an average volumetric gain of 1.05 ± 0.7 cm^3^ [17], no previous studies report the reconstruction of two-wall peri-implant defects with a biological material made of fibrinogen, xenograft, and L-PRF, reinforcing the novelty of this study.

On one hand, the biological input of L-PRF may account for the similar or even slightly superior radiographic outcomes observed in the present study compared with another 12-month prospective study that treated peri-implant crater-like defects using only a bovine-derived xenograft [40] in which the authors reported a statistically significant reduction in bone level from 3.0 ± 0.9 mm to 1.1 ± 0.8 mm.

On the other hand, the impact of L-PRF probably also extends to the significant reductions in PD. Another research group used L-PRF for the treatment of peri-implantitis defects and achieved a six months PD value of 3.30 ± 0.49 mm [41], which is similar to the values here presented, despite the differences between studies regarding PI evaluation periods and peri-implant defect configurations included [42]. However, while Hamzacebi et al. [41] included all types of defects except Cl Ia with a mean initial PD of 6.13 ± 1.05 mm, the present study only included Cl Ib two-wall defects with a mean baseline PD of 8.07 ± 1.51 mm, indicating that a larger effect was obtained (4.73 ± 1.54 mm vs. 2.82 ± 1.03 mm). One possible explanation to this higher value may be the synergistic effect of the xenograft, acting as a scaffold, and the L-PRF matrix with its biological inductive properties [36,37]. This hypothesis is further confirmed by the fact that other authors tested a xenograft with 10% collagen to treat different types of peri-implant defects [29] with no adjunctive use of L-PRF and were unable to achieve PD reductions in Cl Id defects similar to those here presented. It is important to mention, nonetheless, that the authors used a different decontamination method, which could have had an impact on the results.

In fact, implant surface decontamination is a critical step for the success of peri-implantitis reconstructive treatment. In the present study, a glycine air-flow system with a specific nozzle was used, allowing access to the most difficult parts of the implant body [43], particularly the inter-threads. In vitro studies using air-flow devices have yielded promising results, and an air-flow system with glycine powder has been shown to be an efficient option for the debridement of implants with peri-implantitis defects [44,45], representing a valid approach in terms of biocompatibility [46] and also contributing to a beneficial microbiological shift around implants [47]. In a randomized controlled trial aiming to evaluate the efficacy of three mechanical procedures for surgical treatment of peri-implantitis with no defect fill, the single use of an air-flow system using glycine to promote decontamination of peri-implant defects has been demonstrated to significantly reduce bone levels by approximately 1 mm, from 7.34 ± 1.29 mm to 6.44 ± 1.46 mm after 6 months [48]. Despite these promising results, a recent systematic review concluded that no single decontamination method demonstrated clear evidence of superiority compared to others [49], and additional marginal bone gains in surgical approaches may be attributed to graft materials, as demonstrated in a recent systematic review and meta-analysis [10].

Peri-implant health diagnosis requires clinical and radiographic assessment. Probing pocket depths could differ depending on the height of soft tissue at the implant location; however, an increase in PD over time conflicts with peri-implant health. In the present study, we did not have the baseline PD recorded at the time of prosthesis placement. Loss of alveolar bone starting after the implant being placed, in function, should not exceed 2 mm [50]. Unlike PD, this parameter is not position dependent. In the present study, MBL ≥ 3 mm was considered the primary outcome respecting the EFP guidelines and according to some previous systematic reviews and meta-analysis [6,10].

One limitation of the present study was not analyzing patient-centered outcomes like post-operative discomfort and aesthetics. These parameters should be evaluated in future studies. Also, this study has an exploratory nature and serves as a proof-of-concept for the reconstructive treatment of two-wall peri-implant defects using a L-PRF/xenograft block after peri-implant surface decontamination with a glycine air-flow system and implant surface irrigation with L-PRF exudate, demonstrating its potential efficacy and feasibility. For this reason, the anticipated effect size to determine the required number of patients to include in this study is very large and therefore, the number of patients recruited for this prospective study is low, which, associated with the short follow-up period, does not allow generalization of the results. However, these preliminary results should encourage researchers to design further large-scale studies to confirm these findings.

## 5. Conclusions

Within the limitations of this prospective study, the clinical and radiographic results suggest that the proposed surgical combined technique protocol may represent a valid approach for the treatment of non-contained biologically demanding two-wall peri-implant defects.

## Figures and Tables

**Table 1:** Demographic and clinical characteristics of the patients included in this study and characteristics of the dental implants analyzed.

Case	Gender /Age	Smoking Status (Y/N)	Periodontal Disease (Y/N)	Health Status	Implant Position	Years in Function	Prosthesis	Implant (Size/Brand)
#1	M/59 y	N	N	Healthy	14	9	Cemented single unit	4.3 × 10 mm Nobel Replace (Nobel Biocare^®^)
#2	M/37 y	Y (8 c/d)	Y	Healthy	36	11	Screw type single unit	4 × 11.5 mm Branemark MKIII (Nobel Biocare^®^)
#3	F/54 y	N	Y	AntiDep	14	3	Barr overdenture	3.5 × 12 mm essential (Klockner^®^)
#4	M/51 y	N	Y	Healthy	21	6	Screw type single unit	4.1 × 12 mm Bone Level (Straumann^®^)
#5	M/43 y	N	Y	Healthy	11	6	Screw type single unit	4.3 × 16 mm NobelReplace (Nobel Biocare^®^)
#6	F/58 y	N	N	Healthy	23	4	Screw type single unit	4.3 × 13 mm NobelReplace (Nobel Biocare^®^)
#7	F/37 y	N	N	Healthy	11	13	Screw type single unit	3.3 × 13 mm Branemark MK III (Nobel Biocare^®^)
#8	F/56 y	N	Y	Healthy	36	10	Cemented single unit	4.3 × 13 mm NobelReplace (Nobel Biocare^®^)
#9	M/43 y	N	N	Healthy	12	10	Barr overdenture	4.1 × 12 mm Bone Level (Straumann^®^)
#10	F/53 y	N	N	Healthy	12	5	Screw type single unit	3.5 × 13 RePlant (Implant Direct^®^)
Mean (SD)						7.7 (3.16)		

**Table 2:** Mean (Standard deviation) of clinical parameters and marginal bone levels evaluated over the course of 12 months. PD—probing depth; BOP—bleeding on probing; MR—mucosal recession; CAL—clinical attachment loss; SUP—suppuration; KT—keratinized tissue; MBL—marginal bone level. PD, MR, CAL, KT, and MBL in millimeters; BOP and SUP in percentage. Significance obtained with Friedman test.

	Baseline	6 Months	12 Months	*p*
PD (mm)	8.07 (1.51)	3.33 (0.59)	3.53 (0.38)	<0.001
BOP (%)	60.0 (34.0)	13.0 (15.0)	13.0 (13.0)	0.004
MR (mm)	0.00 (0.00)	0.05 (0.08)	0.03 (0.07)	0.097
CAL (mm)	8.07 (1.51)	3.38 (0.62)	3.57 (0.37)	<0.001
SUP (%)	2.0 (5.0)	0.0 (0.0)	0.0 (0.0)	0.368
KT (mm)	4.20 (1.75)	3.40 (1.84)	3.50 (1.78)	0.141
MBL (mm)	5.08 (1.68)	1.58 (0.92)	1.83 (0.90)	0.001

**Table 3:** Variation in the clinical and radiographic parameters between follow-up appointments. PD—probing depth; BOP—bleeding on probing; MR—mucosal recession; CAL—clinical attachment loss; SUP—suppuration; KT—keratinized tissue; MBL—marginal bone level. PD, MR, CAL, KT, and MBL in millimeters; BOP and SUP in percentage. Pairwise comparisons with Bonferroni correction. Significance cut-points: ^ns^—non-significant; *—*p* < 0.01; **—*p* < 0.001.

Parameter	0–6 Months	0–12 Months	6–12 Months
PD	−4.73 (1.54) **	−4.53 (1.30) **	0.20 (0.60) ^ns^
BOP	−55.0 (39.0) *	−55.0 (31.0) **	0.0 (16.0) ^ns^
MR	0.05 (0.08) ^ns^	0.03 (0.07) ^ns^	−0.02 (0.05) ^ns^
CAL	−4.68 (1.51) **	−4.50 (1.28) **	0.18 (0.61) ^ns^
SUP	−2.0 (5.0) ^ns^	−2.0 (5.0) ^ns^	0.0 (0.0) ^ns^
KT	−0.80 (1.40) ^ns^	−0.70 (1.25) ^ns^	0.10 (0.32) ^ns^
MBL	−3.49 (1.17) **	−3.25 (1.32) **	0.25 (0.44) ^ns^

## Data Availability

The authors are available to provide clinical data upon reasonable request.

## References

[1] Jung R.E., Zembic A., Pjetursson B.E., Zwahlen M., Thoma D.S. (2012). Systematic review of the survival rate and the incidence of biological, technical, and aesthetic complications of single crowns on implants reported in longitudinal studies with a mean follow-up of 5 years. Clin. Oral Implant. Res..

[2] Pjetursson B.E., Thoma D., Jung R., Zwahlen M., Zembic A. (2012). A systematic review of the survival and complication rates of implant-supported fixed dental prostheses (FDPs) after a mean observation period of at least 5 years. Clin. Oral Implant. Res..

[3] Albrektsson T., Dahlin C., Jemt T., Sennerby L., Turri A., Wennerberg A. (2014). Is marginal bone loss around oral implants the result of a provoked foreign body reaction?. Clin. Implant Dent. Relat. Res..

[4] Araujo M.G., Lindhe J. (2018). Peri-implant health. J. Clin. Periodontol..

[5] Schwarz F., Derks J., Monje A., Wang H.L. (2018). Peri-implantitis. J. Clin. Periodontol..

[6] Renvert S., Persson G.R., Pirih F.Q., Camargo P.M. (2018). Peri-implant health, peri-implant mucositis, and peri-implantitis: Case definitions and diagnostic considerations. J. Clin. Periodontol..

[7] Derks J., Schaller D., Hakansson J., Wennstrom J.L., Tomasi C., Berglundh T. (2016). Peri-implantitis—Onset and pattern of progression. J. Clin. Periodontol..

[8] Karlsson K., Trullenque-Eriksson A., Tomasi C., Derks J. (2023). Efficacy of access flap and pocket elimination procedures in the management of peri-implantitis: A systematic review and meta-analysis. J. Clin. Periodontol..

[9] Khoshkam V., Del Amo F.S.-L., Monje A., Lin G.H., Chan H.L., Wang H.L. (2016). Long-term Radiographic and Clinical Outcomes of Regenerative Approach for Treating Peri-implantitis: A Systematic Review and Meta-analysis. Int. J. Oral Maxillofac. Implant..

[10] Tomasi C., Regidor E., Ortiz-Vigon A., Derks J. (2019). Efficacy of reconstructive surgical therapy at peri-implantitis-related bone defects. A systematic review and meta-analysis. J. Clin. Periodontol..

[11] Donos N., Calciolari E., Ghuman M., Baccini M., Sousa V., Nibali L. (2023). The efficacy of bone reconstructive therapies in the management of peri-implantitis. A systematic review and meta-analysis. J. Clin. Periodontol..

[12] Castro A.B., Meschi N., Temmerman A., Pinto N., Lambrechts P., Teughels W., Quirynen M. (2017). Regenerative potential of leucocyte- and platelet-rich fibrin. Part B: Sinus floor elevation, alveolar ridge preservation and implant therapy. A systematic review. J. Clin. Periodontol..

[13] Clark R.A. (2001). Fibrin and wound healing. Ann. N. Y. Acad. Sci..

[14] Collen A., Koolwijk P., Kroon M., Van Hinsbergh V.W. (1998). Influence of fibrin structure on the formation and maintenance of capillary-like tubules by human microvascular endothelial cells. Angiogenesis.

[15] van Hinsbergh V.W., Collen A., Koolwijk P. (2001). Role of fibrin matrix in angiogenesis. Ann. N. Y. Acad. Sci..

[16] Bielecki T., Ehrenfest D.M.D., Everts P.A., Wiczkowski A. (2012). The role of leukocytes from L-PRP/L-PRF in wound healing and immune defense: New perspectives. Curr. Pharm. Biotechnol..

[17] Cortellini S., Castro A.B., Temmerman A., Van Dessel J., Pinto N., Jacobs R., Quirynen M. (2018). Leucocyte- and platelet-rich fibrin block for bone augmentation procedure: A proof-of-concept study. J. Clin. Periodontol..

[18] Enkling N., Johren P., Klimberg V., Bayer S., Mericske-Stern R., Jepsen S. (2011). Effect of platform switching on peri-implant bone levels: A randomized clinical trial. Clin. Oral Implant. Res..

[19] Choukroun J., Choukroun J., Adda F., Schoeffler C.V.A., Schoeffler C., Vervelle A. (2001). The opportunity in perio-implantology: The PRF. Implant Dent..

[20] Pinto N., Temmerman A., Teughels W., Castro A., Cortellini S., Quirynen M. (2016). Consensus Guidelines on the Use of L-PRF from the 1st European Meeting on Enhanced Natural Healing in Dentistry. Optimisation of Guided Bone Regeneration Techniques View Project.

[21] Stiesch M., Grischke J., Schaefer P., Heitz-Mayfield L.J.A. (2023). Supportive care for the prevention of disease recurrence/progression following peri-implantitis treatment: A systematic review. J. Clin. Periodontol..

[22] Chan H.L., Lin G.H., Suarez F., MacEachern M., Wang H.L. (2014). Surgical management of peri-implantitis: A systematic review and meta-analysis of treatment outcomes. J. Periodontol..

[23] Monje A., Pons R., Insua A., Nart J., Wang H.L., Schwarz F. (2019). Morphology and severity of peri-implantitis bone defects. Clin. Implant Dent. Relat. Res..

[24] Schwarz F., Sahm N., Schwarz K., Becker J. (2010). Impact of defect configuration on the clinical outcome following surgical regenerative therapy of peri-implantitis. J. Clin. Periodontol..

[25] Polimeni G., Koo K.T., Qahash M., Xiropaidis A.V., Albandar J.M., Wikesjo U.M. (2004). Prognostic factors for alveolar regeneration: Bone formation at teeth and titanium implants. J. Clin. Periodontol..

[26] Wikesjo U.M., Kean C.J., Zimmerman G.J. (1994). Periodontal repair in dogs: Supraalveolar defect models for evaluation of safety and efficacy of periodontal reconstructive therapy. J. Periodontol..

[27] Wikesjo U.M., Selvig K.A. (2000). Periodontal wound healing and regeneration. Periodontology.

[28] Avila-Ortiz G., Bartold P.M., Giannobile W., Katagiri W., Nares S., Rios H., Spagnoli D., Wikesjo U.M. (2016). Biologics and Cell Therapy Tissue Engineering Approaches for the Management of the Edentulous Maxilla: A Systematic Review. Int. J. Oral Maxillofac. Implant..

[29] Roccuzzo M., Gaudioso L., Lungo M., Dalmasso P. (2016). Surgical therapy of single peri-implantitis intrabony defects, by means of deproteinized bovine bone mineral with 10% collagen. J. Clin. Periodontol..

[30] Khoury F., Buchmann R. (2001). Surgical therapy of peri-implant disease: A 3-year follow-up study of cases treated with 3 different techniques of bone regeneration. J. Periodontol..

[31] Roos-Jansaker A.M., Persson G.R., Lindahl C., Renvert S. (2014). Surgical treatment of peri-implantitis using a bone substitute with or without a resorbable membrane: A 5-year follow-up. J. Clin. Periodontol..

[32] Figuero E., Graziani F., Sanz I., Herrera D., Sanz M. (2014). Management of peri-implant mucositis and peri-implantitis. Periodontology 2000.

[33] Dohan D.M., Choukroun J., Diss A., Dohan S.L., Dohan A.J., Mouhyi J., Gogly B. (2006). Platelet-rich fibrin (PRF): A second-generation platelet concentrate. Part I: Technological concepts and evolution. Oral Surg. Oral Med. Oral Pathol. Oral Radiol. Endod..

[34] Dohan D.M., Choukroun J., Diss A., Dohan S.L., Dohan A.J., Mouhyi J., Gogly B. (2006). Platelet-rich fibrin (PRF): A second-generation platelet concentrate. Part II: Platelet-related biologic features. Oral Surg. Oral Med. Oral Pathol. Oral Radiol. Endod..

[35] Schar M.O., Diaz-Romero J., Kohl S., Zumstein M.A., Nesic D. (2015). Platelet-rich concentrates differentially release growth factors and induce cell migration in vitro. Clin. Orthop. Relat. Res..

[36] Chen F.M., Wu L.A., Zhang M., Zhang R., Sun H.H. (2011). Homing of endogenous stem/progenitor cells for in situ tissue regeneration: Promises, strategies, and translational perspectives. Biomaterials.

[37] Roy S., Driggs J., Elgharably H., Biswas S., Findley M., Khanna S., Gnyawali U., Bergdall V.K., Sen C.K. (2011). Platelet-rich fibrin matrix improves wound angiogenesis via inducing endothelial cell proliferation. Wound Repair. Regen..

[38] Strauss F.J., Stahli A., Gruber R. (2018). The use of platelet-rich fibrin to enhance the outcomes of implant therapy: A systematic review. Clin. Oral Implant. Res..

[39] Miron R.J., Zucchelli G., Pikos M.A., Salama M., Lee S., Guillemette V., Fujioka-Kobayashi M., Bishara M., Zhang Y., Wang H.L. (2017). Use of platelet-rich fibrin in regenerative dentistry: A systematic review. Clin. Oral Investig..

[40] Roccuzzo M., Bonino F., Bonino L., Dalmasso P. (2011). Surgical therapy of peri-implantitis lesions by means of a bovine-derived xenograft: Comparative results of a prospective study on two different implant surfaces. J. Clin. Periodontol..

[41] Hamzacebi B., Oduncuoglu B., Alaaddinoglu E.E. (2015). Treatment of Peri-implant Bone Defects with Platelet-Rich Fibrin. Int. J. Periodontics Restor. Dent..

[42] Schwarz F., Herten M., Sager M., Bieling K., Sculean A., Becker J. (2007). Comparison of naturally occurring and ligature-induced peri-implantitis bone defects in humans and dogs. Clin. Oral Implant. Res..

[43] Sahrmann P., Ronay V., Sener B., Jung R.E., Attin T., Schmidlin P.R. (2013). Cleaning potential of glycine air-flow application in an in vitro peri-implantitis model. Clin. Oral Implant. Res..

[44] Petersilka G.J. (2011). Subgingival air-polishing in the treatment of periodontal biofilm infections. Periodontology 2000.

[45] Sahrmann P., Ronay V., Hofer D., Attin T., Jung R.E., Schmidlin P.R. (2015). In vitro cleaning potential of three different implant debridement methods. Clin. Oral Implant. Res..

[46] Toma S., Lasserre J., Brecx M.C., Nyssen-Behets C. (2016). In vitro evaluation of peri-implantitis treatment modalities on Saos-2osteoblasts. Clin. Oral Implant. Res..

[47] Schwarz F., Ferrari D., Popovski K., Hartig B., Becker J. (2009). Influence of different air-abrasive powders on cell viability at biologically contaminated titanium dental implants surfaces. J. Biomed. Mater. Res. B Appl. Biomater..

[48] Toma S., Brecx M.C., Lasserre J.F. (2019). Clinical Evaluation of Three Surgical Modalities in the Treatment of Peri-Implantitis: A Randomized Controlled Clinical Trial. J. Clin. Med..

[49] Baima G., Citterio F., Romandini M., Romano F., Mariani G.M., Buduneli N., Aimetti M. (2022). Surface decontamination protocols for surgical treatment of peri-implantitis: A systematic review with meta-analysis. Clin. Oral Implant. Res..

[50] Lindquist L.W., Carlsson G.E., Jemt T. (1996). A prospective 15-year follow-up study of mandibular fixed prostheses supported by osseointegrated implants. Clinical results and marginal bone loss. Clin. Oral Implant. Res..

